# Clastogenic Factors as Potential Biomarkers of Increased Superoxide Production

**Published:** 2007-12-11

**Authors:** Ingrid Emerit

**Affiliations:** University Paris VI and CNRS France

## Abstract

The formation of clastogenic factors (CF) and their damaging effects are mediated by superoxide, since superoxide dismutase is regularly protective. CF are produced via superoxide and stimulate the production of superoxide by monocytes and neutrophils. This results in a selfsustaining and longlasting process of clastogenesis, which may exceed the DNA repair system and ultimately lead to cancer (Emerit, 1994). An increased cancer risk is indeed observed in conditions accompanied by CF formation. These include irradiated persons, patients with chronic inflammatory diseases, HIV-infected persons and the chromosomal breakage syndromes ataxia telangiectasia, Bloom’s syndrome and Fanconi’s anemia. Biochemical analysis has identified lipid peroxidation products, arachidonic acid metabolites, nucleotides of inosine and cytokines, in particular tumor necrosis factor alpha, as the clastogenic and also superoxide stimulating components of CF. Due to their chromosome damaging effects, these oxidants can be detected with classical cytogenetic techniques. Their synergistic action renders the CF-test particularly sensitive for the detection of a pro-oxidant state. Correlations were observed between CF and other biomarkers of oxidative stress such as decreases in total plasma thiols or increases in TBARS or chemiluminescence. Correlations between CF and disease activity, between CF and radiation exposure, suggest the study of CF for monitoring these conditions. CF may also be useful as biochemical markers and intermediate endpoints for the evaluation of promising antioxidant drugs.

CF formation represents a link between chronic inflammation and carcinogenesis. Prophylactic use of superoxide scavengers as anticarcinogens is therefore suggested.

## Introduction

Clastogenic i.e. chromosome damaging substances are present in the plasma of patients with a variety of pathological conditions accompanied by oxidative stress. These include irradiation exposure, certain chronic inflammatory diseases, HIV infection, ischemia reperfusion injury, as well as the hereditary chromosomal instability syndromes. The formation of these breakage factors or clastogenic factors (CF), as well as their chromosome damaging effects, are mediated by the superoxide anion radical, since they are regularly inhibited by superoxide dismutase (SOD). For this reason, the term “superoxide mediated clastogenesis” has been proposed (Emerit et al. 1996). Superoxide is not a direct damaging agent, but an initiator of a series of events leading to the formation of clastogenic materials. Biochemical analysis of CF preparations identified three major classes of endogenous chemical clastogens: (i) lipid peroxidation products derived from arachidonic acid of membranes, and in particular the highly clastogenic aldehyde 4-hydroxynonenal (Emerit et al. 1991), (ii) cytokines such as tumor necrosis factor alpha (Emerit et al. 1995a), and (iii) unusual nucleotides such as inosine di-and triphosphate (Auclair et al. 1994). The clastogenic properties of these components were confirmed with the respective commercial standards.

In the following the results of our studies on CF formation and CF action, as well as of their significance in the pathogenesis of the above mentioned diseases are summarized.

## Mecanisms of CF Formation and CF Action

The strongest evidence for the role of superoxide in CF formation came from in vitro experiments, in which cells were exposed to superoxide-generating systems, such as a xanthine-xanthine oxidase (X-XO) reaction, a phorbol 12-myristate-13 acetate (PMA) stimulated respiratory burst or photodynamic reactions. The mitoses observed in these cultures showed chromosomal breakage, and the supernatant was clastogenic, when added to other cultures. This could be consistently prevented by addition of SOD (30–150 units/ml). Catalase was only irregularly protective, and hydrogen peroxide was not present in the supernatants at detectable levels. Because it did not seem likely that extracellularly produced superoxide would reach the nucleus without being scavenged by the intracellular SOD abundantly available in the cytosol, we proposed the formation of secondary clastogenic substances as an explanation. Biochemical analysis revealed that CF is not a single factor, but a mixture of chromosome-damaging, pro-oxidant substances. It could also be deduced from these studies that they are cellular products. Indeed, exposure of cell-free culture media or serum did not result in clastogenic ultrafiltrates. After re-suspension of the cells in fresh medium, they continued to release CF in absence of the superoxide-generating system. This was also noted after exposure to irradiation and explains, why plasma of irradiated persons still contains CF during years after the irradiation event.

Our working hypothesis that CF formation is related to lipid peroxidation was confirmed by the detection of metabolites and degradation products of arachidonic acid in CF preparations from X-XO treated cultures. Malondialdehyde and arachidonic acid derived HETES and H(P)ETES are only weak clastogens in our test system, and supernatants become clastogenic only after formation of the breakdown product 4-hydroxynonenal, which is clastogenic at 0.1μM concentration (Emerit et al. 1991). This needs about 18 hours. The clastogenic potential reaches a maximum after 24 h with no further increase thereafter. In PMA- treated cultures, the stimulation of a respiratory burst leads to CF formation via the AA cascade after activation of phospholipase A. TNF alpha is released after activation of protein kinase C. It has been shown that maximal TNF release occurs 20 hours after stimulation, which is similar to the appearance of CF in the culture medium. The clastogenic activity increased linearly with increasing numbers of neutrophils and monocytes in the culture system. CF formation was most important with the strong promotor PMA. The weak promotor 4-0-methyl PMA had only weak clastogenic effects, and the nonpromoting phorbol did not produce CF (Emerit and Cerutti, 1981a).

Photodynamic reactions generate not only singlet oxygen, but may lead also to an oxidized sensitizer and the superoxide anion radical. Chromosome damage and SCEs were induced in blood cultures by addition of either riboflavin or psoralens as photosensitising agents in combination with UVA light of 365 nm wavelength. The supernatants of these cell cultures contained CF, while supernatnts from cultures receiving only the sensitizer or only UVA irradiation were not clastogenic (Alaoui-Youssefiet al. 1994).

Screening of clastogenic HPLC fractions with computerized mass spectroscopy revealed molecular peaks compatible with inosine tri-and diphosphate, nucleotides not produced in the organism under normal conditions (Auclair et al. 1990). Their formation appears to be related to nucleotid pool imbalances, which are secondary to increases in adenosine deaminase and xanthine oxidase. They are not only clastogenic, but have also superoxide stimulating properties, as demonstrated with chemiluminesence or the cytochrome C reduction assay. This leads to amplification of CF formation in the culture system, as this is also the case with TNF.

SOD can prevent CF production, when added before addition of the superoxidizing system or during the first 24 h of the cultivation period. Between 24 and 48 h, the protection is irregular. After 48 h or later, no protection is provided. This is in agreement with the kinetics of CF formation and accumulation of clastogenic products not inhibitable by SOD such as 4-hydroxynonenal for instance. This aldehyde inactivates functional SH groups of DNA polymerases and also forms adducts with cellular thiols (Wavra et al. 1986).

An amplification process is also going on during CF action. In cultures exposed to preformed CF, its components TNF and ITP will generate superoxide and further CF formation. In addition, ITP raises intracellular calcium levels, activates lyosomal enzyme levels and induces chromosome damage through the action of nucleases (Allison and Paton, 1965). In addition, ITP inhibits DNA topoisomerases by competition with the ATP-binding sites of these enzymes (Osheroff et al. 1983). Because of the multifactoriel and multistep process of CF action, addition of CF at the beginning of the cultivation period yields the highest aberration rates. It explains also why the chromosome aberrations are mainly of the chromatid type in cells exposed to CF in the Go phase of their cell cycle. One may deduce that they were not caused immediately upon exposure to CF, but later in the S-phase or in the G2 phase of the cell cycle. Also in agreement with this view is the protective effect of SOD, which is optimal when the enzyme is added 30 min before addition of CF or during the first 24 h. The latter is analogous to the time course of inhibition of CF formation by SOD. Our experiments with fluorescently labelled SOD indicated that the enzyme protects the cells not only by dismutating extracellular superoxide, but that it binds to the cell membrane in particular of monocytes/macrophages (Emerit et al. 1996 and 2002). The superoxide generation by these cells diminishes, and the formation of CF is reduced.

These mechanisms of CF formation and CF action play also a role in the following examples of diseases accompanied by CF.

## Detection of Clastogenic Activity: The CF-Test

The methods for the detection of these endogenous clastogens called CF are the same as those currently used for various exogenous clastogenic agents. Since it is known that the clastogenic activity is in the small molecular weight fraction, the plasma is filtered through Millipore or Amicon ultrafiltration filters. In the initial description of the technique (Emerit, 1990), filters with a cut off at 10,000 daltons were recommended. Afterwards, when TNF was recognized as one of the clastogenic components, filters with a cut off at 30,000 daltons were used. The ultrafiltration step is useful for elimination of all high molecular weight materials, which might disturb culture growth in case of blood group incompatibilities. Also residual cells in patients’ plasma are retained by the filter. CF are unstable at room temperature, and loose their activity overnight in a refrigerator. Frozen, they may be conserved over years. Ultrafiltrates are more stable than serum samples. Repeated freezing and thawing of the samples should be avoided. CF are not inactivated by lyophilization, if done rapidly in small aliquots.

The plasma ultrafiltrates from patients are added to regular blood cultures set up with 0.5 ml of blood from a healthy donor. In case of cytotoxic effects of the usual dose of 250 μl, the culture is repeated with 100 μl. Since the clastogenic effects are related to oxyradical production, a culture medium poor in free radical scavengers is recommended (Keck and Emerit, 1979; Emerit 1990). TCM 199 or RPMI 1640 may be used (5 ml per culture tube), while RPMI 1629 and Ham’s FT are not convenient because of their high L-cysteine content. The serum used for supplementation (1 ml/culture) should have a yellow aspect indicating that there was no hemolysis during its preparation. Fetal calf serum may contain up to 1μg/ml SOD and have anticlastogenic effects in the test system (Baret and Emerit, 1983). Bovine serum may be rich in vitamin E and inhibit clastogenesis related to lipid peroxidation.

Lymphocyte proliferation is stimulated by the addition of phytohemagglutinin M or P. After 48 or 72 h of incubation at 37 °C, the mitoses are arrested in metaphase by addition of colchicine, 2 h before harvesting. It is known from studies with exogenous clastogens that good proliferation of the culture is a prerequisite for evaluation of chromosomal breakage. The mitoses rate is in general better after 72 h, however, if the nature of the chromosomal aberrations and not only the breakage frequencies shall be evaluated, the 48 h cultures are preferred.

Microscopic slides are prepared according to classical cytogenetic procedures, including a hypotonic treatment followed by fixation of the pellet and spreading on wet cold slides. The chromosomes of 50 well-spread and complete metaphase plates are examined on coded slides for the presence of chromatid and isochromatid breaks, telomeric extrusions or acentric fragments. Gaps are not considered as aberrations. Chromosome type aberrations such as rings, dicentrics or other structurally rearranged chromosomes are in general not found after exposure to CF, as already mentioned in connection with CF formation and CF action.

A series of samples is tested the same day on the cultures set up with the same donor’s blood. Two additional cultures without ultrafiltrate serve for the establishment of the spontaneous chromosomal aberration rate of the donor’s lymphocytes. This background level of aberrations is deduced from the aberration rate of the ultrafiltrate-treated cultures of the same donor. The difference of the two values is called the adjusted clastogenic score (ACS). This way of treating results is necessary, since the clastogenic activity of samples collected at subsequent dates will be tested on donors with different background levels of aberrations.

The mean ACS for our laboratory established by the study of 100 ultrafiltrates from plasma of healthy blood donors (Blood Transfusion Center) is 0.8 ± 1.0 percent. Out of the 100 samples, 95 induced no or up to 2 aberrations, only 5 samples resulted in 3 aberrations. According to these criteria, an ACS of +4 (8%) or higher is CF+, an ACS of +3 (6%) is CF±, of 0 or +2 (4%) is CF−.

Probability values of <0.05 are considered to denote significant differences between patients and controls.

Instead of chromosomal aberrations, other endpoints can be studied, such as sister chromatid exchanges (SCE) (Emerit and Cerutti, 1981, Lehnert and Goodwin, 1997), mutations at the HPRT locus (Emerit and Lahoud-Maghani, 1989) or DNA strand breakage. In our experience, chromosomal aberrations are 3–5 times more frequent in CF-treated cultures compared to controls, while SCE rates, though consistently increased, are generally not even doubled. SCE studies are thought to be easier, since they need no cytogenetic training. However, because of the rarety of chromosome type aberrations, the observer may score only open breaks and fragments, which cannot be overlooked.

CF do not induce lesions in isolated DNA. They have to be studied on cellular systems because of the indirect action mechanism of break induction. Fibroblasts, endothelial or mesothelial cells of human origin, or from other species, may be used for CF studies, but show lower breakage rates than blood cultures, in which the presence of monocytes is important. Cultures with pure lymphocytes are not appropriate for CF-studies.

One may also use the appropriate biochemical assays for the various identified components. However, the concentrations of each of the clastogens may not reach detectable levels, while the chromosome damaging effects are the result of the synergistic action of all CF components. The cytogenetic assay is therefore more sensitive.

## Results Obtained with the CF-Test

### Radiation exposure

Chromosome-damaging effects of plasma from therapeutically or accidentally irradiated persons are known since the early seventies (Goh and Sumner, 1968; Hollowell and Littlefield, 1968). Further reports came from A-bomb survivors in Hiroshima (Pant and Kamada, 1977). The existence of radiation-induced CF was confirmed by our laboratory in adults and children exposed as a consequence of the Chernobyl accident. Among 89 liquidators, 37 were positive for CF (41%), in 29% of them, the ACS were +10 and even higher. The number of CF+ individuals was higher among those, for whom the physical dosimetry indicated values exceeding the allowance dose of 25 cGy. Also those, who had been in Chernobyl in 1986 were more often positive for CF than those, who were on site in 1987 and 1988. The percentage of CF positive samples was higher for those working at the reactor site compared to those working elsewhere in the 11–30 km zone. If site of work, data commenced work, duration and type of work, use of protective clothes, changes in blood counts etc were combined as weighting factors, liquidators with a score of >10 weighting factors were more often positive for CF than workers with <10 weighting factors. There was a good correlation between weighting factors and physical dosimetry. Another group of 44 liquidators could be studied after their emigration to Israel. CF positivity was noted in 27% of them despite the fact that they had not been back to Chernobyl since several years and were living now in a non-contaminated area (Emerit et al. 1994, 1995c). Similar observations were made for 170 school children emigrated to Israel (Emerit et al. 1997a). The differences were highly significant between children from Ukraine compared to children emigrated from ‘clean cities’ of the former Soviet union. Highest ACS were found in children from Gomel and Mozyr, which are high exposure sights according to IEAE measurements. Logistic regression analysis revealed a negative association between CF scores and frequency of consumption of fresh vegetables and fruit during the post-immigration period. On the other hand, intake of eggs and fish by boys who were 7 years old or younger prior to immigration was associated with high clastogenic scores (Kordysh et al. 2001).

Irradiation of blood in vitro has shown that doses as low as 50 cGy induce CF. When cells were washed after irradiation and re-suspended in fresh medium, they continued to produce CF. Besides lipid peroxidation products, TNF alpha plays a major role in radiation-induced CF, and TNF mRNA was found to be increased after exposure of cells to ionizing radiation (Hallahan et al. 1989). Even low doses of radiation primed murine peritoneal macrophages for elevated production of TNF (Iwamoto and McBride, 1994), which then stimulates other competent cells for superoxide production. This feedback mechanism may explain the longevity of the clastogenic activity in patients’ plasma (30 years in A-bomb survivors, more than 10 years in liquidators) and be responsible for late effects of radiation such as cancer and leukemia.

According to Lehnert and Goodwin (1997), exposure to alpha particles generates extracellular factor(s) able to cause sister chromatid exchanges in normal human cells. The authors suggest that these diffusable substances are similar to the CF generated by ionizing radiation, and that they are possibly implicated in radon-induced carcinogenesis in the respiratory tract.

Also ultraviolet radiation is able to generate CF by interaction with a photosensitizer (Alaoui-Youssefiet al. 1994). Psoralen plus UVA (PUVA) is one of the treatments proposed for psoriasis. The clastogenic activity increased significantly between the first and the last (16th) exposure to PUVA (Filipe et al. 1997a). CF may contribute to the well-known risk of photo-carcinogenesis following PUVA therapy. UVA and photosensitizers are ubiquitous, therefore CF formation may play also a role in photo-aging of the skin (Filipe et al. 1977) and be responsible for hair graying as a consequence of damage to melanocytes of the hair follicles (Emerit et al. 2004 ).

### Chronic inflammatory diseases

CF formation is not only related to irradiation events, but observed in a variety of chronic inflammatory diseases, where they are also formed via superoxide and stimulate further superoxide production. Epidemiologic studies indicate an increased cancer risk for most of these diseases (Emerit, 1994).

Connective tissue diseases such as *progressive systemic sclerosis* (***PSS***), *systemic lupus erythematosus* (***SLE***), *rheumatoid arthritis* (***RA***) are regularly accompanied by CF formation, an explanation for the so-called “spontaneous” chromosomal instability regularly observed in blood or fibroblast cultures from these patients. Clastogenic activity is found not only in the plasma, but can be isolated also from the supernatants of cultures set up with blood or fibroblasts from these patients. The clastogenic effects are regularly inhibited by SOD.

When clastogenic effects of patients’plasma were studied in a group of 48 ***PSS*** patients, a positive correlation was found between ACS and disease activity (Emerit et al. 1997b). Also high ACS were correlated with an increase of adenosine deaminase (ADA), an ubiquitous enzyme involved in purine metabolism (Meunier et al. 1995). It catalyses the irreversible deamination of adenosine or deoxyadenosine to inosine or deoxyinosine. The increase in ADA leads to the formation of inosine triphosphate (ITP) and inosine diphosphate (IDP). These abnormal nucleotides, have clastogenic and also superoxide stimulating properties inhibitable by SOD (Auclair et al. 1990). They were detected by computerized mass spectrometry in all 12 patients studied, while they were absent in 10 control samples. When ACS, ADA and ITP were studied simultaneously, all three parameters were increased for patients with skin plus internal organ involvement, while neither of them was noted in patients with skin involvement only (Emerit et al. 1997b).

In addition to ADA, the enzyme xanthine oxidase was found to be increased in the plasma of PSS patients (Miesel and Zuber, 1993). This may represent another source of superoxide production via the xanthine-xanthine oxydase system and play a role for scleroderma formation (Murrel, 1993). The causal relationship between oxidative damage and fibrinogenesis is well-known (Poli and Parola, 1997) and explains the progression of sclerosis in PSS. The occurrence of lung and oesophagus cancer is increased on the basis of sclerotic fibrosis (Roumm and Medsger, 1985).

In ***SLE***, clastogenic plasma samples have photosensitizing properties (Emerit and Michelson, 1981). Patients’ lymphocytes are photo-sensitive to light in the near UV wave length, and lymphocytes from healthy persons become also photo-sensitive, when exposed to CF from patients. An increased risk to develop cancer and lymphoma was reported for SLE patients (Petterson et al. 1992).

A high frequency of lymphoreticular neoplasia, associated with increased chromosomal breakage and CF formation, was also observed in the New Zealand Black mouse, the classical animal model for murine lupus. By selective matings according to chromosome breakage frequencies in the bone marrow of the mice, two NZB sublines could be developed (Emerit et al. 1980). At the age of 18 months, the incidence of tumors was 4 times higher in the high breakage (HB) than in the low breakage (LB) line. Both lines differed significantly in spontaneous superoxide production by resident peritoneal macrophages (Khan et al. 1990) and in xenotropic type C virus expression (Bernard and Emerit, 1982). The virus may be responsible for increased superoxide production due to its membrane-active properties. In turn, CF-induced DNA strand breakage may contribute to the release of the endogenous virus. Direct clastogenic action of the virus can be excluded, since it is retained by the ultrafiltration filter during CF isolation from the plasma. Intraperitoneal injection of SOD decreased the chromosome damage in the bone marrow of the treated animals (Emerit et al. 1981).

In ***RA***, plasma and synovial fluid contain CF. Monocytes of these patients are immunologically activated, produce increased amounts of superoxide and release increased amounts of arachidonic acid and TNF alpha. Addition of monocytes from patients to lymphocyte cultures from healthy persons results in clastogenic supernatants. The clastogenic effects of the culture supernatants were correlated with the number of monocytes added. On the other hand, cocultivations of heterologous monocytes from healthy persons did not result in CF. Addition of SOD prevented CF formation (Emerit et al. 1989).

CF formation was also confirmed for inflammatory diseases of the gut such as ulcerative colitis and Crohn’s ileocolitis (Emerit et al. 1979). Cocultivations of mononuclear cells of patients with those of healthy donors resulted in clastogenic supernatants.

Clastogenic activity in the plasma was a regular finding in patients with chronic inflammation of the liver (Emerit et al. 2000; Serejo et al. 2005). Among 40 patients with *chronic hepatitis* C, 37 were positive for CF, mean ACS 9.8 ± 4.4. In another 17 patients, in whom liver cirrhosis had developed, 16 were CF+ with a mean ACS of 11.1 ± 2.7. The highest clastogenic scores were observed with plasma from patients with hepato-carcinoma (12.6 ± 4.2) (Emerit et al 2007). For the majority of these samples, the cultures had to be repeated because of cytotoxic effects with the usual dose of 250 μl. The mechanisms implicated in CF formation are those described in the other inflammatory diseases. The Kupffer cells of the liver, the largest population of macrophages in the human body, release various mediators including superoxide, eicosanoids and cytokines (Jaeschke et al. 1992). High resting levels of superoxide anion were detected in whole blood of hepatitis patients with chemiluminescence studies (Chen et al. 1997), and increased oxyradical production was measured also on liver biopsies with spin trapping techniques (Valgimigli et al. 2000). TNF alpha is produced in excess (Boya et al. 1996). Enhanced levels of xanthine oxidase, and of the substrates xanthine and hypoxanthine are a source of superoxide in virus-infected tissues (Maeda and Akaike, 1991). Also ADA was found to be increased in patients with viral hepatitis (Ungerer et al. 1992), and formation of ITP may result as a consequence of inosine accumulation. Increases in lipid peroxidation products in the plasma and liver of patients with hepatitis C were reported in the literature (DeMaria et al. 1996; Paridis et al. 1997) and noted also in our patients. There was no correlation between malonaldehyde levels and clastogenic scores. This is not astonishing, since the clastogenic effects are not only produced by lipid peroxidation products. However, a trend for higher aminotransferase levels in association with high ACS and MDA levels was noted, when groups and not individuals were considered. Interestingly, high ACS were correlated with necrosis and inflammation in liver biopsies, while fibrosis scores were correlated with MDA levels. An antioxidant preparation containing flavonoids with high superoxide scavenging capacity successfully inhibited CF formation and reduced aminotransferase levels to near normal values (Emerit et al. 2005).

### HIV-infection and AIDS

In a series of 22 ultrafiltrates from HIV-positive plasma, the mean chromosomal aberration rates induced in the test cultures were significantly increased compared to 20 HIV-negative reference samples. CF were already present in asymptomatic seropositive individuals, increased with the progress of the disease to show highest values in patients with Kaposi’s sarcoma. For most samples, the culture had to be repeated with a reduced volume of ultrafiltrate. Antiviral medication had not prevented CF formation in these patients (Fuchs et al. 1995). In addition to their chromosome-damaging effects, CF may play a role in HIV-1 expression. Clastogenic ultrafiltrates upregulated HIV-expression in U1 cells. This promonocytic cell line with two integrated copies of HIV-1 provirus DNA per cell is characterized by a lack of detectable virion production. Exogenous SOD inhibited the clastogenic and the virus-inducing effects of CF (Edeas et al. 1997). HIV-1 expression could be induced in this cell line also by others using a variety of inflammatory cytokines, including TNF-alpha, one of the components of CF (Poli and Fauci, 1992). The virus was not present in the ultrafiltrates, since the filter is retentive for virus particles. The clastogenic effects in the test culture were therefore not due to direct action of the virus on the chromosomes, but induced by the clastogenic components formed via virus-stimulated superoxide production.

In a multiparameter analysis, positive correlations between TNF-alpha and CF, but not with IL-2, IL-6, elastase and neopterin levels were noted. The lipid peroxidation products MDA and hydroxynonenal were increased in the plasma of patients, but there was no significant quantitative difference between patients with moderate and very high clastogenic activity. The high breakage group showed a tendency toward lower concentrations of plasma antioxidants. Glutathione as well as total plasma thiols were decreased, while glutathione peroxidase remained in the normal range. Vitamin C, selenium and zinc were at the lower limit of normal. On the opposite, vitamin A, E and beta carotene were in the normal range in all 22 patients of the study group, who were still in a satisfactory nutritional state (Fuchs et al. 1998).

### Ischemia reperfusion injury

The excessive generation of reactive oxygen species during reperfusion of ischemic organs is thought to be an etiologic factor of tissue injury (McCord, 1985). The sources of oxyradicals include leakage from mitochondria, the xanthine oxidase reaction, arachidonic acid metabolism and release from neutrophils, monocytes, macrophages and endothelial cells. It has been shown that reperfusion accelerates neutrophil chemotaxis to the myocardium and that the invading cells are activated such that they may release a variety of mediators capable of promoting tissue injury (Werns et al. 1988). Direct evidence that oxygen-derived free radicals contribute to post-ischemic myocardial dysfunction was demonstrated in dogs (Bolli et al. 1989).

Our laboratory has studied the formation of CF in ischemia reperfuion injury. In a series of 20 patients undergoing coronary artery bypass grafting, blood samples were taken from the coronary sinus before the aorta was clamped and 20 minutes after myocardial reperfusion was achieved. The blood cultures set up with the blood taken before clamping showed no chromosomal breakage in the dividing lymphocytes of the patients, and the plasma ultrafiltrates induced no chromosomal damage in the test cultures. On the opposite, chromosomal breakage was observed in patients’ cells after twenty minutes of reperfusion, and the plasma ultrafiltrates were clastogenic. The burst of oxyradicals upon reperfusion is probably the initiating event of CF formation which in turn leads to further superoxide generation. This amplification process may explain why detectable levels of CF needed a delay of at least 10 minutes. No CF was found immediately after unclamping. After 20 minutes, samples from peripheral venous blood were also clastogenic, but less than the simultaneous samples taken in the coronary sinus (Emerit et al. 1988 and 1995a). We could also show that the luminol-enhanced chemiluminescence (CL) response of neutrophils from healthy persons was increased when these cells were exposed to CF-containing post-reperfusion samples from patients. Light emission was reduced to normal values in presence of SOD. This was also observed by Kumar et al. (1990), who studied the CL response of the perfused rat heart during ischemia and reperfusion. No light emission was observed in absence of cells, indicating that the CL was due to the respiratory burst of the neutrophils. There was no correlation between clastogenic activity and CL for individual results. However, when groups were formed, low clastogenic activity was correlated with low CL. When the ultrafiltrates of post-reperfusion samples were fractionated by HPLC, chromosome damaging and CL-stimulating activity were eluted in the same range of fractions. The biochemical analysis of the clastogenic fractions was not able to detect ITP, hydroxynonenal or TNF alpha, probably because of the low concentrations of the respective substances in the ultafiltrates.

### Chromosomal breakage syndromes

The chromosomal breakage syndromes ataxia telangiectasia (AT), Bloom’s syndrome (BS) and Fanconi’s anemia (FA) are autosomal recessive disorders, associating chromosomal breakage with a high cancer incidence (German, 1983). Formation of CF could be demonstrated for all three syndromes, but the reasons for increased superoxide production are less evident than for CF formation in irradiated persons, chronic inflammation or ischemia-reperfusion.

In **AT**, a CF was found in patients’ plasma and in conditioned medium from lymphocyte and fibroblast cultures (Shaham and Becker, 1981). Cultured fibroblasts are hypersensitive to agents that induce oxidative stress (Joenje et al. 1987), in particular to the tumor promotor phorbolmyristate acetate (Shiloh et al. 1985).

In **BS**, a first indication for the production by BS fibroblasts of a diffusible component capable of inflicting DNA damage in normal cells came from the work of Tice et al. (1978), who induced sister chromatid exchanges in lymphocyte cultures from healthy subjects with conditioned media from BS fibroblasts. In our laboratory, clastogenic activity was detected in culture media from six BS strains, while normal fibroblast strains yielded negative results (Emerit and Cerutti, 1981b). Concentrated ultrafiltrates of BS culture supernatants also induced SCEs, but with relatively low efficiency compared to chromosomal breakage. Addition of SOD suppressed the chromosome-breaking and SCE-inducing action of CF from BS fibroblasts. We also could isolate CF from the plasma of two BS patients (Emerit et al. 1982). This indicated that the formation of CF is a bona fide characteristic of BS, rather than merely a property of BS fibroblasts in tissue culture. The anticlastogenic action of SOD in cell cultures exposed to CF from patients’ blood again pointed to the role of superoxide radicals as intermediates in the formation of genotoxic effects.

In **FA**, several authors reported that chromosomal breakage was induced in the normal donor cells after bone marrow transplantation to the FA patient (Shaham and Adler, 1986; Miale et al. 1984). Using our experimental conditions, we could isolate CF from the plasma of 12 FA patients. While the plasma ultrafiltrates from homozygotes were regularly clastogenic in the test cultures, those from obligatory heterozygotes were only clastogenic after concentration. The anticlastogenic action of SOD indicated the role of superoxide radicals at the origin of the chromosomal instability caracteristic for FA (Emerit et al. 1995b). Other authors reported oxyradical-related base damage in leukocyte DNA from homozygotes and heterozygotes (Degan et al. 1995), and overproduction of TNF alpha in vitro and in vivo, one of the clastogenic components of CF (Roselli et al. 1994).

## Conclusion

On the basis of the reported data, CF can be considered as biomarkers of a pro-oxidant state. The detection of CF indicates that the chromosomal damage observed in patient’s cells is not transient, but that the person will be exposed to clastogenic effects as long as the vicious circle of CF and superoxide production is not interrupted. The genotoxic effects visible as chromosomal breakage demonstrate that the antioxidant defences and the DNA repair system are overwhelmed. Because superoxide radicals are implicated in CF formation and CF action, antioxidants with superoxide scavenging properties may be protective as anticlastogens and consequently as anticarcinogens. In persons at risk because of their occupation, life style or place of residence, the presence of CF represents an indication for chemoprevention of cancer by antioxidants. Their study is also of interest for the evaluation of promising antioxidant drugs. In addition, the correlations described above for certain chronic inflammatory diseases, between importance of clastogenic activity and disease activity suggest that the CF-test may be useful for monitoring the disease process in these conditions, in particular in patients with chronic hepatitis C, AIDS or PSS. The CF-test may be recommended also for monitoring radiation exposure.

The role of radiation and exogenous DNA damaging chemicals at the origin of cancer is well-known. We here show that also endogenous clastogens are involved in carcinogenesis.
